# Clinical Characteristics of Hydralazine-induced Lupus

**DOI:** 10.7759/cureus.4996

**Published:** 2019-06-25

**Authors:** Homa Timlin, Michael Wu, Monica Crespo-Bosque, Duvuru Geetha, Ashley Ingolia, Uzma Haque, Marilyn C Towns, Thomas Grader-Beck

**Affiliations:** 1 Rheumatology, The Johns Hopkins University School of Medicine, Baltimore, USA; 2 Medicine, The Johns Hopkins University School of Medicine, Baltimore, USA; 3 Internal Medicine, North Oaks Health System, Hammond, USA

**Keywords:** hydralazine, hydralazine induced lupus, hydralazine induced lupus, lupus, lupus, systemic lupus erythematosus (sle), drug induce lupus, drug-induced lupus

## Abstract

Introduction

The use of hydralazine has been associated with the development of lupus erythematosus and lupus-like syndromes. We performed this retrospective study to identify clinical characteristics of individuals who developed hydralazine-induced lupus.

Material and methods

We performed a single-center retrospective review of seven individuals who had a diagnosis of hydralazine-induced lupus by International Classification of Diseases, Ninth Revision (ICD9) code and were on hydralazine prior to their diagnosis. Clinical and laboratory data were obtained from a review of the medical record up to 12-month follow-up.

Results

Of the seven individuals with hydralazine-induced lupus, five were Caucasian (71%) and two were African-American. The mean age at the time of diagnosis was 62 years. Four (57%) were male.

The majority of individuals were exposed to hydralazine for more than 12 months (83%). Four individuals had biopsy-proven lupus nephritis and four individuals had cardiopulmonary and skin involvement. Six patients were positive for antinuclear antibody (ANA) with a homogenous pattern, and five of those were positive for anti-histone antibody. Additionally, positive anti-double-stranded DNA (anti-dsDNA) antibody, anti-cardiolipin antibodies, low complements, positive lupus anticoagulant, and leukopenia were seen in 42% of our cohort. Of the five individuals in whom anti-myeloperoxidase (MPO) antibody was strongly positive, all had renal involvement defined by an elevated creatinine with three having biopsy-proven lupus nephritis. Three other individuals with MPO positivity had concurrent cardiopulmonary and skin involvement. Four individuals were positive for anti-proteinase 3 (PR3) antibody, three of whom were strongly positive with renal involvement defined by an elevated creatinine with two having biopsy-proven lupus nephritis. The level of anti-dsDNA antibody and anti-PR3 antibody normalized at three months while anti-MPO antibody took 12 months to normalize following cessation of hydralazine. When checked, low complement component 3 (C3) and anti-histone antibody persisted past 12 months.

In addition to the withdrawal of hydralazine, six individuals were treated with hydroxychloroquine and five with mycophenolate mofetil. Three of four individuals with renal involvement received plasmapheresis and two received cyclophosphamide and hemodialysis.

Conclusion

Hydralazine can aggravate and unmask incipient lupus. Since the presentation can be varied, early recognition of symptoms is critical. Precautions should be taken before initiating this medication in individuals with certain risk factors. Once diagnosed, potential serological findings such as a positive anti-MPO/anti-PR3 antibody could predict more severe manifestations such as pulmonary-renal complications.

## Introduction

Over 100 drugs have been implicated in drug-induced lupus since its first description in association with sulfadiazine [1]. While drug hypersensitivity can be triggered by relatively low or transient doses of the inciting agent after sensitization, the probability of expressing drug-induced autoantibodies and symptomatic lupus increases as the dose and duration of exposure increases [2].

The most common medications causing systemic drug-induced lupus are hydralazine (7%-13%), procainamide, isoniazid, minocycline, and tumor necrosis factor-a (TNF-a) inhibitors [3-5]. 

Several mechanisms for drug-induced lupus have been proposed, including genetic predisposition, drug biotransformation, epigenetic dysregulation, and promotion of neutrophil extracellular traps (NETs). The NET formation is triggered by neutrophil muscarinic receptors in vitro, which demonstrates the potential contribution of the innate immune response in the development of drug-induced lupus [6].

Hydralazine, a potent vasodilator, was first introduced as an antihypertensive agent in 1952. Since then, many cases of hydralazine-induced lupus have been reported. After the publication of the African American Heart Failure Trial (A-HeFT) in 2014, the use of hydralazine increased [7]. Given this increase, physicians should be aware of the signs and symptoms of drug-induced lupus so that prompt discontinuation of the drug can occur.

We performed this retrospective study to identify individuals with hydralazine-induced lupus and to describe their clinical and serological outcomes over time. Common serologies seen in drug-induced lupus were evaluated, including anti-histone antibody and antinuclear antibody (ANA). Less frequent serologies such as anti-proteinase 3 (PR3), anti-myeloperoxidase (MPO) and anti-glomerular basement membrane (GBM) antibodies were also analyzed due to the higher percentage of our cohort having pulmonary renal manifestations.

## Materials and methods

Methods

In this single-center retrospective study, individuals were identified by International Classification of Diseases Ninth Revision (ICD9) codes for hydralazine-induced lupus and who were on hydralazine prior to diagnosis. The Office of Human Subjects Research and Institutional Review Board approved this study.

Acquisition of clinical and laboratory data

Individual demographics, clinical features, dose and duration of hydralazine exposure and details of immunosuppressive therapy were obtained retrospectively from the electronic medical record. Laboratory data including complete blood counts, dilute Russell's viper venom time (dRVVT), ANA, anti-histone antibody, anti-double-stranded DNA (anti-dsDNA) antibody, anti-Smith antibody, anti-ribonucleoprotein (RNP) antibody, anti-cardiolipin antibodies, anti-beta 2-glycoprotein antibodies, anti-MPO antibody, anti-PR3 antibody, Coombs, and serum complement component 3 and 4 (C3, C4) levels were recorded. Multiple time points were evaluated (T=0, 3 months, 6 months, and 12 months); however, data was not always collected based on the availability of results.

Study definitions

Antibodies were considered positive if the ANA level was ≥ 1:160 and the anti-dsDNA antibody level was twofold above the reference range. Proteinuria was defined by urine protein to creatinine ratio of >0.2 g/Cr. Other organ involvement such as lung, heart, and skin were defined by history, imaging or a diagnostic biopsy.

## Results

Of the seven individuals with hydralazine-induced lupus, five were Caucasian (71%) and two were African-American; four (57%) were male. Ages ranged from 28 to 78 years with a mean age of 62 years. The majority of individuals were exposed to hydralazine for more than 12 months (83%) (Table 1).

**Table 1:** Demographics M: male; F: female; W: white; AA: African-American; bid: twice daily; tid: three times daily; qid: four times daily; NA: not available

Subject number	Age	Gender	Race	Hydralazine dose	Hydralazine exposure duration
1	28	M	AA	50 mg tid	More than 12 months
2	78	F	W	100 mg tid	More than 12 months
3	74	M	W	75 mg bid	More than 12 months
4	71	M	W	100 mg tid	More than 12 months
5	52	F	W	100 mg tid	Less than 12 months
6	58	M	W	100 mg tid	More than 12 months
7	74	F	AA	NA	NA

Six of the seven individuals were positive for a homogenous pattern ANA which ranged from a titer of 1:640 to 1:1280. Of these six individuals, five were positive for anti-histone antibody. Notably, all individuals were negative for anti-Smith antibody and anti-RNP antibody. Positive anti-dsDNA antibody, anti-cardiolipin antibodies, low complements, positive lupus anticoagulant, and leukopenia were seen in 42% of our cohort (Table 2).

**Table 2:** Serological features at presentation Y: Yes; N: No; NA: not available; ANA: antinuclear antibody; anti-Sm: anti-Smith antibody; anti-dsDNA: anti-double stranded antibody; anti-RNP: anti-ribonucleoprotein antibody; C3: complement component 3; C4: complement component 4; ACL: anti-cardiolipin; IgM: immunoglobulin M; IgG: immunoglobulin G; IgA: immunoglobulin A; C-ANCA: cytoplasmic antineutrophil cytoplasmic antibodies; P-ANCA: perinuclear antineutrophil cytoplasmic antibodies; anti-MPO: anti-myeloperoxidase antibody; anti-PR3: anti-protease 3; anti-GBM: anti-glomerular basement membrane

Subject number	ANA	anti-histone	anti-Sm	anti-dsDNA	anti-RNP	Low C3	Low C4	Lupus anticoagulant	ACL IgM	ACL IgG	ACL IgA	anti-B2 Glycoprotein (IgA, IgM, IgG)	C-ANCA	P-ANCA	Anti-MPO	Anti-PR3	Anti-GBM
1	N	Y	N	N	N	N	N	N	N	Low positive	N	N	NA	NA	NA	NA	NA
2	1:1280	NA	N	Y	N	Y	Y	NA	NA	NA	NA	NA	N	Y (2.8)	NA	NA	N
3	1:640	Y	N	Y (1:80)	N	N	N	N	Low positive	Low positive	Low positive	N	Y (1:40)	Y (strong positive)	Y (strong positive)	Y (weak positive)	NA
4	1:640	Y	N	N	N	Y	Y	Y	Low positive	N	N	N	N	Y (1:160)	Y (strong positive)	Y (strong positive)	N
5	1:640	Y	N	Y (1:20)	N	N	N	Y	Low positive	Low positive	N	N	N	Y (1:640)	Y (strong positive)	Y (strong positive)	N
6	1:640	Y	N	N	N	Y	N	Y	N	N	N	N	N	Y (>1:1280)	Y (strong positive)	Y (strong positive)	NA
7	1:640	Y	N	N	N	N	N	N	N	N	N	N	NA	Y (>1:640)	Y (strong positive)	N	NA

In regards to hematologic manifestations, one individual had a positive Coomb’s test, one had a positive antiplatelet antibody with persistent thrombocytopenia for up to six months, six were lymphopenic, and all were anemic (Table 3).

**Table 3:** Hematologic features at presentation Y: Yes; N: No; NA: not available

Subject number	leukopenia <4	absolute lymphopenia	elevated absolute eosinophils	anemia	thrombocytopenia	positive anti-platelet antibody	positive Coombs
1	N	N	N	Y	N	NA	NA
2	N	Y	N	Y	N	NA	NA
3	Y	Y	N	Y	N	NA	Y
4	N	Y	N	Y	Y	Y	NA
5	Y	Y	N	Y	N	NA	N

Of the five individuals in whom anti-MPO antibody was strongly positive, all had renal involvement defined by an elevated creatinine with three having biopsy proven lupus nephritis. Three other individuals with anti-MPO antibody positivity had concurrent cardiopulmonary and skin involvement. Four individuals were positive for anti-PR3 antibody, and three of them were strongly positive with renal involvement defined by an elevated creatinine with two having biopsy proven lupus nephritis.

Two individuals had an elevated anti-dsDNA antibody and two had low complement. Three patients had strongly positive anti-MPO antibody and two had strongly positive anti-PR3 antibody.

After hydralazine withdrawal, the level of anti-dsDNA antibody and anti-PR3 antibody was observed to normalize at three months while anti-MPO antibody normalized at six months. Low C3 and anti-histone antibody persisted at 12 months. Low positive anti-cardiolipin antibody normalized at six months.

One individual, who was already on hemodialysis, had a negative ANA and improved with discontinuation of hydralazine without the need for immunosuppression.

In addition to the withdrawal of hydralazine, six individuals were treated with hydroxychloroquine, and five with mycophenolate mofetil. Four patients received pulse dose steroids. Three of the four individuals with renal involvement received plasmapheresis, two required initiation of hemodialysis and two received cyclophosphamide (Table 4).

**Table 4:** Treatment Y: Yes; N: No; NA: not available; IV: intravenous; MMF: mycophenolate mofetil; AZA: azathioprine; CTX: cyclophosphamide; PLEX: plasmapheresis; RTX: rituximab; HD: hemodialysis; HCQ: hydroxychloroquine

Subject number	Prednisone	IV steroid	MMF	AZA	CYC	PLEX	RTX	HD	HCQ
1	N	N	N	N	N	N	N	Y, started prior	N
2	Y	Y	Y	N	Y	Y	N	N	Y
3	Y	N	N	N	N	N	N	N	Y
4	Y	Y	Y	N	N	Y	N	Y	Y
5	Y	NA	Y	N	N	N	N	N	Y
6	Y	Y	Y	N	N	Y	N	N	Y
7	Y	Y	Y	N	Y	N	N	Y	Y

## Discussion

While there are no definitive tests or criteria for the diagnosis of drug-induced lupus, proposed guidelines have aided physicians in making this diagnosis [3,8]. In our study, drug-induced lupus was defined by hydralazine initiation prior to a formal diagnosis of lupus based on the 2012 Systemic Lupus International Collaborating Clinics (SLICC) classification criteria.

Hypothesized risk factors linked to hydralazine-induced lupus include high daily doses of hydralazine, hydralazine therapy longer than three months, slow acetylators, Human Leukocyte Antigen- DRw4 (HLA-DRw4) phenotype, female gender and a family history of autoimmune disease [9-11]. Those receiving lower doses of hydralazine or rapid acetylators, however, are not free from the risk of developing drug-induced lupus [12-14].

Individuals can develop a variety of signs and symptoms after one month of hydralazine therapy but commonly have undergone treatment for years. Hydralazine-induced lupus should be suspected when an individual taking this medication presents with some combination of arthralgias (commonly in the hands and wrists), myalgias, fever, rash, and/or serositis [12-13]. In severe cases, the syndrome may be accompanied by life-threatening manifestations such as vasculitis, glomerulonephritis, or pulmonary failure [11-17].

The main serological findings in drug induced lupus are ANA positivity (90%-100%), anti-histone antibody positivity (90%-95%) and hematological abnormalities (anemia, leukopenia, and positive Combs). These findings are not specific to drug-induced lupus and are shared with idiopathic lupus, therefore making them nonspecific. Rare findings include positive anti-dsDNA antibody, anti-Smith antibody, anti- Sjogren's Syndrome related antigen A (SSA), and MPO/PR3 antibody [16]. 

In particular, for hydralazine-induced lupus, Rubin et al. identified several unique laboratory features such as thrombocytopenia (<5%), hypocomplementemia (<5%), positive anticardiolipin antibodies (5%-15%), anemia (35%) and leukopenia (5%-25%). Additionally, over 95% of study participants had a positive ANA and anti-histone antibody [14].

Although renal involvement is believed to be uncommon in hydralazine-induced lupus, Shapiro et al. studied six individuals with immune complex glomerulonephritis after treatment with hydralazine of 50-300 mg per day for a period of six months to seven years. These individuals were also noted to have anemia (100%), hypocomplementemia (50%), positive ANA (100%) and antibodies to double-stranded DNA antibody (66%) [17].

In our series of seven individuals with hydralazine-induced lupus, several laboratory findings were in line with previous studies with the majority of our study participants having been on therapy for >12 months and had positive ANA and positive anti-histone antibodies. Interestingly, the positive anti-histone antibody persisted up to 12 months after withdrawal of hydralazine.

Other notable laboratory abnormalities, including hypocomplementemia, were seen in three individuals in our series with only one individual having a persistently low C3 at 12 months post withdrawal of hydralazine. Additionally, hematologic abnormalities were also seen in our cases of drug-induced lupus with three individuals presenting with leukopenia, six individuals presenting with lymphopenia, and all individuals presenting with anemia. Only one individual had a positive antiplatelet antibody with persistent thrombocytopenia at six months.

Interestingly when tested, anti-MPO and anti-PR3 antibodies seem to correlate with individuals with pulmonary renal disease thus revealing a potentially important prognostic association. Furthermore, after the withdrawal of hydralazine, anti-MPO antibody positivity seem to persist for six months while anti-PR3 antibody positivity persisted for only three months.

While a potentially important prognostic indicator for individuals who develop hydralazine-induced lupus, our retrospective study is limited by a small sample size, short follow up, and inconsistent serological testing. We hope with further studies this potential association can be further validated and long-term outcomes can be better assessed.

## Conclusions

Hydralazine can aggravate and unmask incipient lupus and persist even after withdrawal of the causative agent. Severe cases may require corticosteroids and other immunosuppressive therapies. Therefore, understanding the risk factors of drug-induced lupus will help risk stratify individuals who have an underlying predisposition for drug-induced lupus. Studies have indicated a possible serological profile in drug-induced lupus, which was also supported by our cohort.

## References

[1] Hoffman BJ (1945). Sensitivity to sufadizine resembling acute disseminated lupus erythematosus. Arch Derm Syphilol.

[2] Woosley RL, Drayer DE, Reidenberg MM, Nies AS, Carr K, Oates JA (1978). Effect of acetylator phenotype on the rate at which procainamide induces antinuclear antibodies and the lupus syndrome. N Engl J Med.

[3] Vasoo S (2006). Drug-induced lupus: an update. Lupus.

[4] Borchers A, Keen C, Gershwin M (2007). Drug-induced lupus. Ann N Y Acad Sci.

[5] Zamorano A, Pedrera L, Lozano C (2010). Drug-induced lupus [Article in English, Spanish]. Med Clin (Barc).

[6] Irizarry-Caro JA, Carmona-Rivera C, Schwartz DM, Khaznadar SS, Kaplan MJ, Grayson PC (2018). Brief report: drugs implicated in systemic autoimmunity modulate neutrophil extracellular trap formation. Arthritis Rheumatol.

[7] Taylor A, Ziesehe S, Yancy C (2004). Combination of isosorbide dinitrate and hydralazine in blacks with heart failure. N Engl J Med.

[8] Xiao X, Chang C (2014). Diagnosis and classification of drug-induced autoimmunity (DIA). J Autoimmun.

[9] Harland S, Faccini V, Timbrell J (1980). Hydralazine-induced lupus erythematosus-like syndrome in a patient of the rapid acetylator phenotype. Br Med J.

[10] Cameron H, Ramsey L (1984). The lupus syndrome induced by hydralazine: a common complication with low dose treatment. Br Med J.

[11] Adams L, Mongey A (1994). Role of genetic factors in drug-related autoimmunity. Lupus.

[12] Tetikkurt C (2016). Drug-induced lupus syndrome. J Vasc.

[13] Finks S, Finks A, Self T (2006). Hydralazine-induced lupus: maintaining vigilance with increased use in patients with heart failure. South Med J.

[14] Rubin RL (2005). Drug-induced lupus. Toxicology.

[15] Olsen N (2004). Drug-induced autoimmunity. Best Pract Res Clin Rheumatol.

[16] Timlin H, Liebowitz J, Jaggi K, Geetha D (2018). Outcomes of hydralazine induced renal vasculitis. Eur J Rheumatol.

[17] Shapiro K, Pinn V, Harrington JT, Levey AS (1984). Immune complex glomerulonephritis in hydralazine-induced SLE. Am J Kidney Dis.

